# Urinary Perchlorate and Thyroid Hormone Levels in Adolescent and Adult Men and Women Living in the United States

**DOI:** 10.1289/ehp.9466

**Published:** 2006-10-05

**Authors:** Benjamin C. Blount, James L. Pirkle, John D. Osterloh, Liza Valentin-Blasini, Kathleen L. Caldwell

**Affiliations:** Division of Laboratory Sciences, National Center for Environmental Health, Centers for Disease Control and Prevention, Atlanta, Georgia, USA

**Keywords:** exposure, iodine, NHANES, perchlorate, thyroid, thyroxine, TSH

## Abstract

**Background:**

Perchlorate is commonly found in the environment and known to inhibit thyroid function at high doses. Assessing the potential effect of low-level exposure to perchlorate on thyroid function is an area of ongoing research.

**Objectives:**

We evaluated the potential relationship between urinary levels of perchlorate and serum levels of thyroid stimulating hormone (TSH) and total thyroxine (T_4_) in 2,299 men and women, ≥ 12 years of age, participating in the National Health and Nutrition Examination Survey (NHANES) during 2001–2002.

**Methods:**

We used multiple regression models of T_4_ and TSH that included perchlorate and covariates known to be or likely to be associated with T_4_ or TSH levels: age, race/ethnicity, body mass index, estrogen use, menopausal status, pregnancy status, premenarche status, serum C-reactive protein, serum albumin, serum cotinine, hours of fasting, urinary thiocyanate, urinary nitrate, and selected medication groups.

**Results:**

Perchlorate was not a significant predictor of T_4_ or TSH levels in men. For women overall, perchlorate was a significant predictor of both T_4_ and TSH. For women with urinary iodine < 100 μg/L, perchlorate was a significant negative predictor of T_4_ (*p* < 0.0001) and a positive predictor of TSH (*p* = 0.001). For women with urinary iodine ≥ 100 μg/L, perchlorate was a significant positive predictor of TSH (*p* = 0.025) but not T_4_ (*p* = 0.550).

**Conclusions:**

These associations of perchlorate with T_4_ and TSH are coherent in direction and independent of other variables known to affect thyroid function, but are present at perchlorate exposure levels that were unanticipated based on previous studies.

Perchlorate is an inorganic anion used for a variety of products such as road flares, explosives, pyrotechnics, and solid rocket propellant (Mendiratta et al. 1996). Perchlorate can also form naturally in the atmosphere, leading to trace levels of perchlorate in precipitation (Dasgupta et al. 2005). Natural processes are considered to concentrate perchlorate in some locations such as regions of west Texas (Dasgupta et al. 2005) and northern Chile (Urbansky et al. 2001). A combination of human activities and natural sources has led to the widespread presence of perchlorate in the environment. As of November 2005, perchlorate was detected in drinking water samples from 4.1% of community water supplies in 26 different states, with levels ranging from the method detection limit of 4 μg/L to a maximum at 420 μg/L [U.S. Environmental Protection Agency (EPA) 2005]. Most of this drinking-water contamination is likely due to contaminated source waters, although in rare instances perchlorate formation has been reported to occur in water distribution systems (Jackson et al. 2004). Additionally, perchlorate exposure from the diet is probable because of the contamination of milk (Kirk et al. 2005), vegetables (Sanchez et al. 2005), fruit (Sanchez et al. 2006a), grain (Sanchez et al. 2006b), and forage crops (Jackson et al. 2005). Perchlorate contamination has also been reported in dietary supplements and flavor enhancers (Snyder et al. 2006).

Trace levels of perchlorate in the environment leads to human exposure. Direct measurement of perchlorate in biological samples collected from people [National Research Council (NRC) 2005] is considered an excellent assessment of their exposure. We recently assessed perchlorate exposure in a nationally representative sample of 2,820 U.S. residents, ≥ 6 years of age, who participated in the National Health and Nutrition Examination Survey (NHANES) during 2001 and 2002 (Blount et al. 2006).

Environmental perchlorate exposure is of potential health concern because much larger doses of perchlorate have been shown to competitively inhibit iodide uptake (Greer et al. 2002; Wyngaarden et al. 1953). Populations with low intake of iodine or increased demand for iodine may be more vulnerable to inhibition of iodide uptake. Sustained inhibition of iodide uptake can lead to hypothyroidism, although perchlorate-induced changes to thyroid function have not been previously demonstrated in any human population exposed to perchlorate, even at doses as high as 0.5 mg/kg body weight per day (NRC 2005). The thyroid plays a crucial role in energy homeostasis and neurologic development. Hypothyroidism can lead to metabolic problems in adults and abnormal development during gestation and infancy (Braverman and Utiger 2000). Severe hypothyroidism due to iodine deficiency during pregnancy is a preventable cause of cretinism, a permanent cognitive impairment of the developing fetus (Glinoer 2000). Mild hypothyroidism during pregnancy has been associated with subtle cognitive deficits in children (Haddow et al. 1999; Klein et al. 2001), leading the NRC to recommend that consideration be given to adding iodide to all prenatal vitamins (NRC 2005). Therefore, we examined relationships between urinary perchlorate and serum thyroid hormones in men and women, ≥ 12 years of age, who participated in NHANES 2001–2002.

## Subjects and Methods

### Study design

NHANES is conducted by the National Center for Health Statistics of the Centers for Disease Control and Prevention (CDC). This survey is designed to assess the health and nutrition status of the civilian, non-institutionalized U.S. population. NHANES uses a complex multistage probability sampling designed to be representative of the U.S. population based on age, sex, race/ethnicity, and income. Data reported in the present study were collected using an extensive household interview addressing health conditions and health-related behaviors and a standardized physical examination including medical blood and urine tests, which were conducted in mobile examination centers. NHANES 2001–2002 was conducted in 30 locations throughout the United States. Overall, the survey interview response rate was 83.9% and the exam response rate was 79.6%. A full description of the NHANES survey is available on the NHANES website (CDC 2004). The study protocol was reviewed and approved by the CDC institutional review board; additionally, informed written consent was obtained from all subjects before they took part in the study.

Urinary perchlorate levels were measured by the Division of Laboratory Sciences, National Center for Environmental Health, on a representative random one-third subsample consisting of 2,820 study participants (males and females), ≥ 6 years of age (Blount et al. 2006). For ages ≥ 12 years, 2,517 persons were in the random subsample. Serum levels of thyroid stimulating hormone (TSH) and total thyroxine (T_4_) were only available for 2,299 participants ≥ 12 years of age.

### Demographic variables

Sociodemographic data were self-reported by study participants. Race/ethnicity was derived from self-reported questionnaire data and categorized as non-Hispanic white, non-Hispanic black, Mexican American, and “other.” Each of these race/ethnicity categories was used in the regression modeling. Non-Hispanic whites were used as the referent group in regression analysis.

### Laboratory methods

During the physical examinations, whole blood and spot urine specimens were collected from participants, aliquoted, and stored cold (2–4°C) or frozen until shipment. Whole blood was collected into a red-top 15-mL Vacutainer tube, mixed, allowed to clot 30–45 min, and centrifuged; approximately 1 mL serum was stored frozen in a cryovial for future analysis for TSH and T_4_. Serum samples collected in 2001 were assayed for TSH and T_4_ by the Coulston Foundation (Alamogordo, NM) using a microparticle enzyme immunoassay for the quantitative determination of TSH, and a Hitachi 704 chemistry analyzer (Hitachi Chemical Diagnostics, Mountain View, CA) for the quantitative determination of T_4_ (CDC 2003). Serum samples collected in 2002 were assayed for TSH and T_4_ by Collaborative Laboratory Services (Ottumwa, IA) using a chemiluminescent immunoassay (Access Immunoassay System; Beckman Instruments, Fullerton, CA) (CDC 2003). The National Center for Health Statistics evaluated the TSH and T_4_ data sets from the two laboratories and determined that the values are comparable across the 2 years.

Surplus urine samples from NHANES 2001–2002 were shipped on dry ice to the Division of Laboratory Sciences and analyzed for perchlorate, thiocyanate, and nitrate using ion chromatography tandem mass spectrometry (Blount et al., 2006; Valentin-Blasini et al. 2005). These samples were stored frozen (−70°C) for up to 4 years before perchlorate analysis. Experiments evaluating storage at −70°C for > 2 years indicated no changes in urinary levels of this analyte (Blount et al. 2006). Reported results for all assays met the division’s quality control and quality assurance performance criteria for accuracy and precision [similar to specifications outlined by Westgard et al. (1981)]. Urine samples from the same study participants had previously been analyzed for iodine using inductively coupled plasma mass spectrometry (Caldwell et al. 2005).

### Statistical analysis

Initial multiple regression analysis found perchlorate to be a significant predictor of both T_4_ and log TSH in women, but perchlorate did not predict either T_4_ or log TSH in men (data not shown). Therefore, we present subsequent analysis focused on women.

Of the 1,318 women ≥ 12 years of age, 92 had missing TSH and T_4_ values, leaving 1,226. Of these 1,226 women, 91 were excluded from analysis because they reported a history of thyroid disease or current use of thyroid medications, leaving 1,135 women. Of these 1,135 women, 3 had extreme values of T_4_ and/or TSH and were excluded. One of these women had a total T_4_ of 27 μg/dL and a TSH of 0.04 IU/L. This woman was clearly hyperthyroid and thus was excluded from the analysis. Two other women had very high TSH levels (43 and 68 IU/L) and were excluded. Of the remaining 1,132 women, 21 had missing perchlorate measurements, leaving a sample size of 1,111 women.

The major design variables for NHANES are age, sex, race/ethnicity, and income related to the poverty level. The values of these variables for the initial 1,318 women and the final 1,111 women, respectively, are as follows: mean age, 41.6 and 39.8 years; percent non-Hispanic whites, 70.8% and 69.4%; percent non-Hispanic blacks, 11.8% and 12.5%; percent Mexican Americans, 7.0% and 7.0%; and percent below the poverty level, 13.9% and 14.9%.

We chose covariates for the multiple regression analyses that are known to be or likely to be associated with T_4_ or TSH. We selected a broad number of covariates to evaluate the independence of the perchlorate relationship. These covariates were age, race/ethnicity, body mass index (BMI), serum albumin, serum cotinine (a marker of tobacco smoke exposure), estimated total caloric intake, pregnancy status, postmenopausal status, premenarche status, serum C-reactive protein, hours fasting before sample collection, urinary thiocyanate, urinary nitrate, and use of selected medications.

For these covariates, Table 1 provides means (or geometric means if lognormally distributed) for continuous variables, percent in category for categorical variables, and number of missing results for each covariate. Thyroid function has been previously reported to vary with the constitutional variables of age, race, sex, pregnancy, and menopause (Braverman and Utiger 2000). Serum cotinine is a marker of tobacco smoke exposure, and smoking is associated with altered thyroid function (Bertelsen and Hegedus 1994). We included serum C-reactive protein as a marker for inflammatory conditions that have been associated with alterations in thyroid function. Both total caloric intake [based on a 24-hr dietary recall survey and a U.S. Department of Agriculture (USDA) database (Food and Nutrition Database for Dietary Studies; USDA 2004)] and BMI are related to thyroid function, but the interrelationship as to cause or effect is unclear.

Serum albumin was included in our analysis as a possible surrogate for T_4_ serum protein binding. NHANES 2001–2002 included total T_4_ measurements but not free T_4_ measurements; total T_4_ varies with the concentrations of specific binding proteins. Concentrations of these proteins can change with physiologic state and health conditions. Free T_4_ varies less with such protein concentration changes than does total T_4_. Serum albumin accounts for 15–20% of T_4_ binding, with thyroid binding protein and prealbumin (not measured in NHANES) accounting for the remaining percentage (Robbins 2000). Thyroid autoantibody measurements were not available for 2001–2002. For autoantibodies to affect the relationship between perchlorate and T_4_ or TSH, presence of autoantibodies would have to correlate with perchlorate levels. We have found no such correlation in the literature and we are unaware of a rationale for such an association.

Medications known to affect thyroid function were also considered. As noted above, women taking medication containing thyroid hormone (e.g., levothyroxine) or antithyroid drugs (e.g., methimazole or propylthiouracil) were excluded. Use of beta-blockers, estrogen formulations, steroids, and furosemide were each modeled using an indicator variable in the regressions. An “other drug” category was also modeled by an indicator variable. This “other drug” category consisted of a heterogeneous group of other medications that have possible effects on thyroid function, protein binding, or measurements, including salicylates, dopaminergics, anticonvulsants and barbiturates, narcotic analgesics, androgenic agents, lithium, and several others (a total of 28 drug codes).

We included the log of urinary creatinine in the models to adjust for variable water excretion. A nonlinear relationship was evaluated by adding the square of the log of perchlorate to final models, but it was not significant. Models were also checked for significance of interaction terms involving main effects. We examined partial regression plots to identify any unduly influential data points; no unduly influential points were found. Indicator variable coefficients in the models (e.g., for non-Hispanic blacks) were interpreted as follows: 1 = group member, and 0 = not a group member. Urine samples were collected in three sessions of the day from 0800 hours through 2200 hours. Mean perchlorate levels were not statistically different across sessions (*p* = 0.49).

We examined univariate statistics and distribution plots for each dependent and independent variable to look for outliers and to assess the distribution shape. TSH, perchlorate, cotinine, BMI, urinary thiocyanate, urinary nitrate, and C-reactive protein were log_10_-transformed to normalize their distributions.

Regression models, including log of perchlorate as one of the predictor variables, were constructed separately for thyroxine and log of TSH. For the initial phase of analysis, we used ordinary least-squares regression (OLS) (SAS Proc Reg, version 9.0; SAS Institute, Cary, NC) and purposefully did not adjust for the NHANES complex survey design in order to obtain a broad group of potentially significant predictor variables. Forward stepwise and backward elimination procedures were used on both population-weighted and unweighted data. The entry *p*-value for forward elimination models was 0.10 and the retaining *p*-value for backward elimination was 0.10 in order to identify significant and borderline significant predictors. The forward stepwise and backward elimination approaches produced models that were generally in good agreement.

This OLS analysis produced a generous list of significant and borderline-significant variables for regression analysis using SUDAAN (version 9.0.1; Research Triangle Institute, Research Triangle Park, NC), which provides an analysis that adjusts for the complex survey design. SUDAAN regression models were tested using a manual backward elimination approach starting with the variables obtained from the OLS regression modeling. Selected variables that were excluded in the SUDAAN backward elimination process were added to the final model to ensure they were not significant. The stability of the perchlorate coefficient was monitored during the SUDAAN backward elimination process.

In the main SUDAAN regression analysis, we used population weights to represent women ≥ 12 years of age in the U.S. population for the years 2001 and 2002. In addition, we performed separate regression analyses with SUDAAN using unweighted data and verified that regression coefficients were in good agreement with those obtained using population weights. Reported regression model results in the tables use the population-weighted analysis.

Women were categorized based on a urinary iodine cut point of 100 μg/L and analyzed separately. The 100 μg/L cut point was used based on the World Health Organization (WHO) definition of sufficient iodine intake in populations (WHO 1994). The WHO noted that the prevalence of goiter begins to increase in populations with median urinary iodine < 100 μg/L. A urine iodine level of 100 μg/L represents about the 36th percentile of urinary iodine concentrations in women living in the United States (Caldwell et al. 2005). Women with lower iodine intake could be more vulnerable to perchlorate’s effects to impair iodine uptake. From this analysis, the significance of urinary perchlorate as a predictor of thyroid function in women was found to be largely determined by women with urinary iodine < 100 μg/L. Consequently, we report here results for women divided into groups based on urinary iodine levels.

Compared to the use of average multiple spot urine measurements or 24-hr urine specimens, the use of a single spot urine for perchlorate and iodine measurement has more imprecision in estimating true urine levels (Andersen et al. 2001). This imprecision is a source of random error (not bias) and therefore decreases statistical power to detect an association between perchlorate and either TSH or T_4_ compared to these other urine collection approaches.

## Results

For all women ≥ 12 years of age, multiple regression analysis found urinary perchlorate to be a significant predictor of serum TSH and a significant predictor of serum T_4_ (data not shown). Because low iodine levels had potential to affect the relationship of perchlorate with T_4_ and TSH, women with urinary iodine < 100 μg/L were analyzed separately from women with urinary iodine ≥ 100 μg/L. Results of this analysis are presented in Tables 2 and 3 for T_4_ and in Tables 4 and 5 for TSH.

For women with urinary iodine < 100 μg/L, multiple regression analysis found perchlorate to be a significant predictor (*p* < 0.0001) of T_4_ with a coefficient for log perchlorate of −0.8917. The result of regression of T_4_ on perchlorate and urinary creatinine without other covariates yielded a coefficient of −0.8604 (*p* < 0.0001). Perchlorate was also a significant predictor (*p* = 0.0010) of log TSH with a coefficient of 0.1230. The result of regression of log TSH on perchlorate and urinary creatinine without other covariates found a coefficient of 0.1117 (*p* = 0.0031). The signs of these coefficients are coherent, with increased perchlorate associated with less production of T_4_ and an increase in TSH to stimulate additional T_4_ production. For women with urinary iodine ≥ 100 μg/L, perchlorate was not a significant predictor of T_4_ (*p* = 0.5503) but remained a significant predictor of log TSH (*p* = 0.0249). The regression analysis results in Tables 2–5 include variables that were borderline significant (0.05 ≤ *p* < 0.10) to give ample opportunity for other variables to explain variance and better evaluate the independence of the perchlorate effect.

Regression results for men (not shown) indicated that perchlorate was not a significant predictor of either T_4_ or log TSH. This finding also held when examining men with urinary iodine levels < 100 μg/L.

From the regression coefficients for women with urinary iodine < 100 μg/L, we calculated the predicted effect size (i.e., the change in T_4_ and TSH) for different levels of perchlorate exposure. We chose perchlorate levels corresponding to the 5th, 10th, 25th, 50th, 75th, 90th, and 95th percentiles of urinary perchlorate in women ≥ 12 years of age. The minimum and maximum perchlorate values are observed results for this population sample; they are not estimates of the 0th and 100th percentiles for the U.S. population. As such, they would be expected to change in another population sample. The effect size was calculated from the difference between the minimum level of perchlorate measured in women and the level of perchlorate corresponding to the specific percentile. For example, the 50th percentile of urinary perchlorate for women was 2.9 μg/L and the minimum level was 0.19 μg/L. Increasing exposure from 0.19 μg/L to 2.9 μg/L would result in a predicted decrease in T_4_ of 1.06 μg/dL.

For TSH, one more step is needed in the calculation. Because TSH was modeled as log TSH, the change in TSH from a given change in perchlorate depends on the starting level of TSH. In our calculations we used the approximate 50th and 90th percentiles of TSH as starting points to estimate the predicted perchlorate effect size for TSH. Results of these calculations for T_4_ and TSH are presented in Table 6. For comparison, the normal range is 5–12 μg/dL for T_4_ and 0.3–4.5 IU/L for TSH.

To search for a threshold for the perchlorate relationship with T_4_ and TSH, piecewise regression models (Neter et al. 1985) were fit to the data. No inflection point was found for the perchlorate relationship with T_4_ or TSH. However, statistical power is limited to detect such a threshold, if present.

## Discussion

Increased urinary perchlorate was associated with increased TSH and decreased T_4_ for women with urinary iodine levels < 100 μg/L, a group possibly more susceptible to competitive inhibition of thyroid iodine uptake by perchlorate. The statistically significant associations of urinary perchlorate with decreased serum T_4_ and increased serum TSH were consistent with competitive inhibition of iodide uptake.

For women with urine iodine ≥ 100 μg/L, perchlorate was also a statistically significant predictor for TSH but not for T_4_. Greater iodine intake may have diminished the effect of perchlorate on T_4_ in these women. The significant association with TSH, but not with T_4_, in this group may be due to the greater sensitivity of TSH to impairment of thyroid function; that is, normal T_4_ levels are maintained by increasing TSH to compensate for impaired thyroid function.

Predicted changes in serum TSH and T_4_ with increasing perchlorate exposure (Table 6) can span a notable portion of the normal medical range of TSH and T_4_ values. Compared with a urine level of 0.19 μg/L, urinary perchlorate of 13 μg/L (95th percentile) yields a predicted decrease in T_4_ of 1.64 μg/dL. The normal range for T_4_ is 5–12 μg/dL. A similar exposure would increase TSH by 2.12 IU/L for a woman starting with a TSH level of 3.11 IU/L (90th percentile for TSH in women ≥ 12 years of age). The normal range for TSH is 0.3–4.5 IU/L. Effect size estimates that start with the 90th percentile of TSH have more uncertainty than estimates starting with the 50th percentile because the predicted TSH levels fall further from the central portions of the original data.

The mechanism of perchlorate’s effect is competitive inhibition of iodide uptake by the thyroid (Clewell et al. 2004; Wolff 1998). Based on this mechanism, individuals with less iodide available to compete with perchlorate may be more vulnerable to impaired iodide uptake. Chronically impaired iodide uptake could lead to changes in serum thyroid hormones, consistent with the increased TSH and decreased T_4_ we find associated with increased perchlorate exposure in women with urinary iodine < 100 μg/L. The WHO (2004) has identified median urinary iodine levels ≥ 100 μg/L as indicating sufficient iodine intake for a population. Based on concerns about adequate iodine intake, the NRC (2005) recently recommended that consideration be given to adding iodine to all prenatal vitamins.

In the present study, perchlorate was not found to be a significant predictor of T_4_ or TSH in men. Previous studies report that women have a much higher risk of goiter than do men, especially in populations with marginal iodine intake (Laurberg et al. 2000). The increased vulnerability of women may partially be caused by increased susceptibility to auto-immune thyroid disease in women, the increased demands on the thyroid during pregnancy, or the effect of estrogens on thyroid function. Estradiol has been shown to block TSH-induced sodium/iodide symporter (NIS) expression in the FRTL5 rat follicular cell line (Furlanetto et al. 1999). Impaired NIS expression could lead to reduced ability of the thyroid follicular cells to import iodide, and thus an increased vulnerability to NIS-inhibitors such as perchlorate. Also, estrogens increase T_4_-binding globulin and thus increase the demand for T_4_ so that free T_4_ levels can remain constant.

Covariates in the regression models predicted T_4_ and TSH levels in a manner generally consistent with previous studies. We found that estrogen use was a significant, independent, and positive predictor of T_4_ in both low and sufficient iodine models of women ≥ 12 years of age, but was not a significant predictor in either of the TSH models. Similar to estrogen use, pregnancy was a significant or borderline significant predictor of T_4_ but not TSH. Both estrogen use and pregnancy raise estrogen levels, increase thyroid binding proteins, and increase serum T_4_ concentrations (Glinoer 1997). Menopause lowers estrogen levels and was a significant predictor of T_4_ in the regression for women with urinary iodine levels < 100 μg/L.

In NHANES III (1988–1994), non-Hispanic blacks were reported to have lower TSH than other groups, and Mexican Americans had higher T_4_ levels than non-Hispanic blacks and whites (Hollowell et al. 2002). The models for TSH and T_4_ in the present study were consistent with these previous findings concerning race/ethnicity. Non-Hispanic blacks have also been shown to have lower urinary perchlorate levels than non-Hispanic whites, although the reason for this difference is not known (Blount et al. 2006). Age was positively associated with TSH in women with urinary iodine levels ≥ 100 μg/L, but not significant for women with urinary iodine levels < 100 μg/L. A positive association of age and TSH was seen in NHANES III and other studies (Canaris et al. 2000; Hollowell et al. 2002).

BMI was significant in the TSH model for women with urinary iodine levels ≥ 100 μg/L, and total caloric intake was significant in the T_4_ model for women with urinary iodine levels < 100 μg/L. Thyroid function clearly has an effect on BMI, as seen clinically and documented in populations (Nyrnes et al. 2006). The reverse is also true, because BMI and total caloric intake can influence the hypothalamic–pituitary–thyroidal axis, although usually at the extremes of body weight and caloric intake (Acheson et al. 1984; Burger et al. 1987; Danforth et al. 1979; Loucks et al. 1992; Loucks and Heath 1994). Total caloric intake in NHANES is a 24-hr recall of food intake. Depending on how well recent intake reflects long-term intake, total caloric intake may parallel the effect of BMI, which was not seen in the present study. Increased caloric intake is known to increase thyroid hormone disposition through deiodination pathways (Burger et al. 1987; Danforth et al. 1979), increasing the conversion of T_4_ to the active form, triiodothyronine (T_3_), and increasing conversion of T_3_ to inactive forms. The effect of changes in calories and carbohydrate composition of the diet on thyroid disposition may have different short- and long-term effects on T_3_ and T_4_ levels. In the present study, hours of fasting before sample collection was a borderline significant predictor in one regression model: T_4_ in women with sufficient iodine. Fasting for 60 hr can reduce TSH in humans, but fasting for shorter periods has unknown effects on thyroid function.

Beta-blocker drugs are commonly used to treat hypertension and other cardiovascular conditions. Beta-blockers inhibit the conversion of T_4_ to the more active form, T3, and increase serum TSH (Kayser et al. 1991). Use of these drugs was positively associated with TSH in the regression for women with urinary iodine < 100 μg/L. Serum C-reactive protein was positively associated with T_4_ in women in each of the iodine groups. C-reactive protein is an acute phase reactant protein increased in many inflammatory conditions in response to production of tissue-generated cytokines, particularly interleukin-6, and has been used as a marker for both specific and systemic low-level inflammation conditions. It is unclear if C-reactive protein is associated with thyroid function other than thyroiditis (Jublanc et al. 2004; Pearce et al. 2003; Tuzcu et al. 2005). However, the stimulus for C-reactive protein, interleukin-6, has a firm inverse relationship with serum T_3_ in nonthyroidal illnesses. Also, C-reactive protein and serum T_4_ binding proteins are synthesized by the liver; C-reactive protein may vary with an unrecognized health or physiologic condition that affects the synthesis of both proteins. The association of C-reactive protein and T_4_ in our study is unclear.

Other variables that are known to possibly affect thyroid function or measurements were not significant predictors in the regression models, including the categories of medications (other than estrogen use and beta-blockers), serum albumin, and serum cotinine. Generally, other medication categories were small and unlikely to have significant effects. Serum albumin did not appear in the final models. Factors such as estrogen use that increase protein binding of thyroid hormones may have accounted for variance in T_4_ due to protein binding that serum albumin may have otherwise explained. Serum cotinine is a marker of tobacco smoke exposure, and smoking is associated with altered thyroid function (Belin et al. 2004; Bertelsen and Hegedus 1994). However, tobacco smoke also contains other factors that can inhibit TSH secretion (Bartalena et al. 1995), and perhaps is an explanation for the absence of an association of serum cotinine with either TSH or T_4_.

Cyanide in tobacco smoke is metabolized to thiocyanate, a competitive inhibitor of iodide uptake (Tonacchera et al. 2004). Also, nitrate from dietary sources and from formation by intestinal bacteria can compete with iodide. *In vitro* studies indicate that perchlorate is a more potent inhibitor of human NIS, with potencies 15, 30, and 240 times greater than thiocyanate, iodide, and nitrate, respectively (Tonacchera et al. 2004). Thus, the ability of NIS to transport adequate amounts of iodide depends on the relative concentrations of these competing anions. Based on the relative concentrations of perchlorate, nitrate, and thiocyanate likely to be found in human serum, several researchers have predicted that nitrate and thiocyanate are more likely than perchlorate to impair thyroid function (DeGroef et al. 2006; Gibbs 2006). Thiocyanate-induced NIS inhibition is a plausible explanation of the association of smoking with goiter in populations with low iodine intake (Knudsen et al. 2002) and is analogous to the association of perchlorate exposure with thyroid hormone levels observed in our study. However, in women with urinary iodine levels ≥ 100 μg/L, urinary thiocyanate was negatively associated with serum TSH, a direction unexpected based on a mechanism of NIS inhibition. The explanation for this is unclear. Urinary nitrate was negatively associated with serum T_4_ in women with urinary iodine levels ≥ 100 μg/L, a direction consistent with inhibition of NIS. Goitrogenic effects of nitrate intake in animal studies have been observed (Wyngaarden et al. 1953), but there are few studies in humans.

Recently the NRC (2005) evaluated the potential health effects of perchlorate ingestion. Based on studies of long-term treatment of hyperthyroidism and clinical studies of healthy adults, the NRC panel estimated that a perchlorate dose of > 0.40 mg/kg/day would be required to cause hypothyroidism in adults, although lower doses may lead to hypothyroidism in sensitive subpopulations (NRC 2005).

Comparison of our results to previous studies requires consideration of *a*) target population group studied, *b*) estimated dose of perchlorate, *c*) duration of exposure to perchlorate dose, and *d*) sample size (statistical power). First, for men, we found no relationship with perchlorate and T_4_ or TSH. This finding is in general agreement with predicted effects of this level of perchlorate exposure based on reported studies of exposure in men. Lawrence et al. (2000) administered 10 mg perchlorate daily (~ 0.14 mg/kg) to iodine-sufficient adult males for 14 days and found a 10% decrease in radioactive iodine uptake (RAIU), but with no change in TSH or free T_4_.

Greer et al. (2002) administered perchlorate to 16 male and 21 female volunteers for 14 days, and found increasing RAIU inhibition for doses between 0.02 and 0.5 mg/kg/day, with no perchlorate-related change in TSH or free T_4_. An unknown number of women in that study may have had urinary iodine < 100 μg/L, but if the women were typical of the U.S. population (Caldwell et al. 2005), the predicted number of women with low urinary iodine would be 7–8. Braverman et al. (2006) administered perchlorate to 13 iodine-sufficient male and female volunteers at daily doses of 0.5 mg and 3 mg for 6 months, and found no change in RAIU, TSH, or free T_4_. Two other studies have also found that workers exposed to perchlorate intermittently for long periods did not have significant changes to serum TSH or T_4_ levels (Braverman et al. 2005; Lamm et al. 1999). These study populations were either exclusively (Braverman et al 2005) or predominantly (Lamm et al 1999) male.

For women, only two perchlorate studies have focused on women or included a large percentage of women. A recent study of 184 pregnant Chilean women, with mean urinary perchlorate levels near the 99th percentile for women in NHANES 2001–2002, found no perchlorate relationship with thyroid function (Tellez et al. 2005). Of these 184 women, 181 had mean urinary iodine levels ≥ 100 μg/L and only 3 had mean levels < 100 μg/L. Therefore, the results of Tellez et al. (2005) would compare to the present results for women with urinary iodine levels ≥ 100 μg/L. Urinary iodine levels in the Chilean study population (median 269 μg/L) were higher than urinary iodine levels found in the NHANES 2001–2002 population [median 168 μg/L; 95% confidence interval, 159–178 μg/L]. The Chilean women (Tellez et al. 2005) were also pregnant, which increases the variability in T_4_ and TSH. This increased variability would make an association between perchlorate and thyroid function harder to find. The second study with a large percentage of women was Greer et al. (2002) discussed above. These two studies are compared with the present study in Table 7.

Table 7 indicates that our study is the first to target and separately analyze results for women with lower levels of urinary iodine, a potentially susceptible population. A second special attribute of the present study is the much larger sample size of women, affording more statistical power to detect a potential effect. By averaging over many women, the current data likely represents a good approximation of a population steady-state exposure to perchlorate that women have had for a long period of time. If a mid- to long-term exposure is needed for perchlorate to affect thyroid function, this data would have a better opportunity to detect that effect than study designs using short-term exposures. The influence of duration of exposure merits further study.

Accurate assessment of exposure is critical to detect biochemical end points potentially related to exposure. Our laboratory recently developed an improved method for measuring urinary perchlorate, which enhances individual perchlorate exposure assessment (Valentin-Blasini et al. 2005). The use of this new urinary perchlorate measurement strengthens the ability of the present study to detect potential associations with T_4_ and TSH.

The present study has the general limitations of a cross-sectional analysis. Therefore, the relationship between urinary perchlorate and thyroid function was examined with attention to the potential influences of chance, bias, or confounding. Perchlorate (as with any of the significant predictor variables) could be a surrogate for another unrecognized determinant of thyroid function. We also assumed in this analysis that urinary perchlorate correlates with levels in the thyroid stroma and tissue, a kinetically distinct compartment. This would be the case in a population with stable, chronic exposures, which is likely but not certain in this population. A large sample size helps to average such potential kinetic differences. Finally, a measurement of free T_4_ would be an improvement to the study.

## Conclusions

Urinary perchlorate is associated with an increased TSH and decreased total T_4_ in women ≥ 12 years of age with urine iodine levels < 100 μg/L in the U.S. population during 2001–2002. For women with urine iodine levels ≥ 100 μg/L, urine perchlorate is a significant predictor of TSH but not T_4_. These effects of perchlorate on T_4_ and TSH are coherent in direction and independent of other variables known to affect thyroid function, but are found at perchlorate exposure levels that were unanticipated based on previous studies. Further research is recommended to affirm these findings.

## Figures and Tables

**Table 1:** Means and percent in category for covariates used in the multiple regression, women ≥ 12 years of age, NHANES 2001–2002.^a^

Variable	No.	No. missing	Arithmetic mean (95% CI)	Geometric mean (95% CI)	Percent in category (95% CI)
Age (years)	1,111	0	39.8 (38.1–41.6)		
Fasting (hr)	1,111	0	10.4 (9.85–10.9)		
Serum albumin (g/dL)	1,111	0	4.20 (4.17–4.23)		
Serum T_4_ (μg/dL)	1,111	0	8.27 (7.97–8.58)		
Total kilocalories (kcal/1,000)	1,072	39	1.93 (1.87–1.99)		
BMI	1,075	36		25.8 (25.2–26.5)	
Serum cotinine (μg/L)	1,104	7		0.33 (0.23–0.48)	
Serum C-reactive protein (mg/dL)	1,111	0		0.16 (0.14–0.18)	
Serum TSH (IU/L)	1,111	0		1.36 (1.31–1.42)	
Urine creatinine (mg/dL)	1,109	2		81.4 (76.7–86.5)	
Urine iodine (μg/L)	1,111	0		126 (115–138)	
Urine nitrate (μg/L × 1,000)	1,106	5		38.0 (35.9–40.3)	
Urine perchlorate (μg/L)	1,111	0		2.84 (2.54–3.18)	
Urine thiocyanate (μg/L × 1,000)	1,104	7		1.20 (1.08–1.33)	
Race/ethnicity					
Non-Hispanic white	1,111	0			69.4 (62.9–75.4)
Non-Hispanic black	1,111	0			12.5 (7.49–19.1)
Mexican American	1,111	0			7.02 (5.14–9.34)
Other race	1,111	0			11.1 (7.04–16.3)
Medication usage					
Furosemide	1,111	0			1.99 (1.25–3.01)
Glucocorticoids and androgens	1,111	0			2.23 (1.24–3.67)
Beta-blocker	1,111	0			4.48 (3.34–5.87)
Estrogen	1,111	0			17.1 (13.2–21.7)
Other drug	1,111	0			1.04 (0.52–1.88)
Menopausal or postmenopausal	1,028	83			35.9 (30.1–41.9)
Pregnant	1,111	0			3.84 (2.74–5.21)
Premenarchal	1,019	92			1.06 (0.48–2.02)

CI, confidence interval.

a Excludes women with missing TSH, T_4_, or perchlorate, women with history of thyroid disease or taking thyroid drugs, and three women with outlier values of T_4_ or TSH (see text).

**Table 2:** Regression of serum T_4_ on perchlorate and covariates for women ≥ 12 years of age with urine iodine < 100 μg/L, NHANES 2001–2002.

Independent variable	Coefficient	SE	*p*-Value
Intercept	8.6508	0.5428	< 0.0001
Log (urinary perchlorate)	−0.8917	0.1811	< 0.0001
Log (urinary creatinine)	0.6897	0.3338	0.0391
Estrogen use	1.5117	0.4421	0.0007
Log (C-reactive protein)	0.8249	0.1774	< 0.0001
Mexican American^a^	0.6296	0.3684	0.0878
Menopause	−0.5908	0.2578	0.0221
Pregnant (by test)	0.7389	0.3662	0.0439
Total kilocalorie intake (÷ 1,000)	−0.3334	0.1173	0.0046
Premenarche	0.6401	0.2722	0.0189

Dependent variable: serum T_4_ (*n* = 348; *R*^2^ = 0.240).

a Referent group for race is non-Hispanic white.

**Table 3:** Regression of serum T_4_ on perchlorate and covariates for women ≥ 12 years of age with urine iodine ≥ 100 μg/L, NHANES 2001–2002.

Independent variables	Coefficient	SE	*p*-Value
Intercept	10.6652	1.2345	< 0.0001
Log (urinary perchlorate)	0.2203	0.3687	0.5503
Log (urinary creatinine)	1.3138	0.7183	0.0677
Estrogen use	0.8278	0.2722	0.0024
Log (C-reactive protein)	0.5783	0.1247	< 0.0001
Mexican-American^a^	0.5763	0.2522	0.0225
Pregnant (by test)	1.6175	0.3334	< 0.0001
Log (urinary nitrate)	−1.1215	0.4994	0.0249
Hours of fasting	0.0290	0.0156	0.0630

Dependent variable: serum T_4_ (*n* = 724; *R*^2^ = 0.149).

a Referent group for race is non-Hispanic white.

**Table 4:** Regression of serum TSH on perchlorate and covariates for women ≥ 12 years of age with urine iodine < 100 μg/L, NHANES 2001–2002.

Independent variables	Coefficient	SE	*p*-Value
Intercept	0.2654	0.1183	0.0403
Log (urinary perchlorate)	0.1230	0.0373	0.0010
Log (urinary creatinine)	−0.0954	0.0761	0.2103
Beta-blocker use	0.1881	0.0595	0.0016
Estrogen use	−0.0918	0.0404	0.0233
Premenarche	0.1288	0.0262	< 0.0001

Dependent variable: log of serum TSH (*n* = 356; *R*^2^ =0.061).

**Table 5:** Regression of serum TSH on perchlorate and covariates for women ≥ 12 years of age with urine iodine ≥ 100 μg/L, NHANES 2001–2002.

Independent variables	Coefficient	SE	*p*-Value
Intercept	−0.6948	0.3415	0.0600
Log (urinary perchlorate)	0.1137	0.0506	0.0249
Log (urinary creatinine)	−0.1198	0.0910	0.1884
Age in years	0.0025	0.0006	< 0.0001
Log (BMI)	0.4812	0.1346	0.0004
Non-Hispanic black^a^	−0.1125	0.0335	0.0008
Log (urinary nitrate)	0.1087	0.0591	0.0660
Log (urinary thiocyanate)	−0.0816	0.0352	0.0206

Dependent variable: log of serum TSH (*n* = 697; *R*^2^ = 0.145).

a Referent group for race is non-Hispanic white.

**Table 6:** Predicted change in serum T_4_^a^ and serum TSH^b^ levels based on changes in urinary perchlorate levels in women ≥ 12 years of age, with urine iodine < 100 μg/L, NHANES 2001–2002.

		Change in TSH (IU/L)^c^	
Change in urine perchlorate^d^	Change in T_4_ (μg/dL)	Initial TSH of 1.40 IU/L (50th TSH percentile)	Initial TSH of 3.11 IU/L (90th TSH percentile)
0.19 to 0.65 μg/L (5th percentile)	0.48	0.23	0.51
0.19 to 0.92 μg/L (10th percentile)	0.61	0.30	0.67
0.19 to 1.6 μg/L (25th percentile)	0.83	0.42	0.93
0.19 to 2.9 μg/L (50th percentile)	1.06	0.56	1.24
0.19 to 5.2 μg/L (75th percentile)	1.28	0.70	1.56
0.19 to 9.0 μg/L (90th percentile)	1.49	0.85	1.89
0.19 to 13 μg/L (95th percentile)	1.64	0.95	2.12
0.19 to 100 μg/L (maximum)	2.43	1.63	3.61

a Normal range for T_4_: 5–12 μg/dL.

b Normal range for TSH: 0.3–4.5 IU/L.

c Depends on initial TSH level.

d Minimum level measured, 0.19 μg/L.

**Table 7:** Comparison of perchlorate studies targeting women or including a high percentage of women.

	Greer et al. (2002)	Tellez et al. (2005)	Present study
No. of females studied	21 (37 total subjects)	184	1,111
No. of females with urine iodine < 100 μg/L	Unknown (estimate 7–8)	3^a^	348, T_4_ analysis 356, TSH analysis
Females with urine iodine < 100 μg/L analyzed separately	No	No	Yes
Perchlorate dose and duration of exposure	Up to 0.5 mg/kg/day for 14 days	Long-term environmental exposure	Long-term environmental exposure
Comments		All women pregnant, increasing variability of T_4_ and TSH	

a Average of one to three spot urine samples.
